# Magnetic Resonance-Guided Laser Interstitial Thermal Therapy for Management of Low-Grade Gliomas and Radiation Necrosis: A Single-Institution Case Series

**DOI:** 10.3390/brainsci12121627

**Published:** 2022-11-28

**Authors:** Lea Scherschinski, Jubran H. Jubran, Kelly A. Shaftel, Charuta G. Furey, Dara S. Farhadi, Dimitri Benner, Benjamin K. Hendricks, Kris A. Smith

**Affiliations:** 1 Department of Neurosurgery, Barrow Neurological Institute, St. Joseph’s Hospital and Medical Center, Phoenix, AZ 85013, USA; 2 Department of Neurosurgery, Charité—Universitätsmedizin Berlin, 10117 Berlin, Germany

**Keywords:** low-grade glioma, oligodendroglioma, astrocytoma, laser interstitial thermal therapy, LITT, minimally invasive, survival

## Abstract

Background: Laser interstitial thermal therapy (LITT) has emerged as a minimally invasive treatment modality for ablation of low-grade glioma (LGG) and radiation necrosis (RN). Objective: To evaluate the efficacy, safety, and survival outcomes of patients with radiographically presumed recurrent or newly diagnosed LGG and RN treated with LITT. Methods: The neuro-oncological database of a quaternary center was reviewed for all patients who underwent LITT for management of LGG between 1 January 2013 and 31 December 2020. Clinical data including demographics, lesion characteristics, and clinical and radiographic outcomes were collected. Kaplan–Meier analyses comprised overall survival (OS) and progression-free survival (PFS). Results: Nine patients (7 men, 2 women; mean [SD] age 50 [16] years) were included. Patients underwent LITT at a mean (SD) of 11.6 (8.5) years after diagnosis. Two (22%) patients had new lesions on radiographic imaging without prior treatment. In the other 7 patients, all (78%) had surgical resection, 6 (67%) had intensity-modulated radiation therapy and chemotherapy, respectively, and 4 (44%) had stereotactic radiosurgery. Two (22%) patients had lesions that were wild-type *IDH1* status. Volumetric assessment of preoperative T1-weighted contrast-enhancing and T2-weighted fluid-attenuated inversion recovery (FLAIR) sequences yielded mean (SD) lesion volumes of 4.1 (6.5) cm^3^ and 26.7 (27.9) cm^3^, respectively. Three (33%) patients had evidence of radiographic progression after LITT. The pooled median (IQR) PFS for the cohort was 52 (56) months, median (IQR) OS after diagnosis was 183 (72) months, and median (IQR) OS after LITT was 52 (60) months. At the time of the study, 2 (22%) patients were deceased. Conclusions: LITT is a safe and effective treatment option for management of LGG and RN, however, there may be increased risk of permanent complications with treatment of deep-seated subcortical lesions.

## 1. Introduction

Low-grade gliomas (LGG) comprise a heterogeneous group of slow-growing tumors of neuroectodermal origin, which includes astrocytic, oligodendrocytic, and ependymal tumor subtypes [1,2]. The World Health Organization (WHO) classifies LGG as grade I and grade II tumors of the central nervous system [1,2,3,4]. Advances in molecular tumor biology have permitted a refined stratification of LGG into one that combines the insights of growth behavior with novel prognostic molecular markers, including *IDH1/2* mutation, 1p/19q codeletion, and *ATRX* mutation, amongst others [1,2,3,4].

Low-grade gliomas account for 6.5% of primary brain tumors, with an estimated annual incidence of 0.46 per 100,000 individuals for astrocytoma and 0.23 for oligodendroglioma [5,6]. Predominantly diagnosed between the fourth and fifth decade of life, LGG have a 5-year survival prognosis of 50% for diffuse astrocytoma and 81% for oligodendroglioma [1,6]. Treatment decision-making for recurrent LGG is nuanced, weighing the advantages of symptom-alleviating tumor reduction against the potential for treatment-related complications.

Magnetic resonance-guided laser interstitial thermal therapy (LITT) has emerged as a minimally invasive treatment modality that employs heat liberated from a laser device to focally ablate diseased tissue [7,8]. As such, LITT obviates the short-term (fatigue) and long-term (radiation necrosis (RN), cognitive disability, secondary malignancies) radiation toxicity commonly observed with stereotactic radiosurgery (SRS) and intensity-modulated radiation therapy (iMRT) [9,10]. As compared to open surgical resection, LITT is associated with a potentially reduced morbidity rate [11], which has resulted in broad expansion of its application for primary and recurrent gliomas, brain metastases, RN, hypothalamic hamartomas, and epilepsy [7,12,13,14,15,16]. In view of these features, LITT is particularly compelling for the treatment of patients with lesions that are deemed inoperable, and those with a high operative risk profile or with prior failed treatments.

While progression-free survival (PFS) analyses for default treatment options such as surgical resection (5.5 to 6.2 years) [17,18], SRS (3.4 to 5.3 years) [19,20,21,22], and iMRT [23] have been studied largely, survival outcomes after LITT treatment in LGG remain limitedly available at this time [24,25]. Only one study presented by Leonardi et al. evaluated survival outcomes after LITT in a larger-scale adult case series comprising 7 recurrent low-grade astrocytomas, reporting a mean overall survival (OS) of 144 months after diagnosis and 34 months after LITT, and a mean PFS of 16 months after LITT [24]. In the pediatric population, Pehlivan et al. assessed LITT as a safe and efficient treatment option in 4 children with LGG whose seizure expressions significantly improved [25]. In the same study, patients with LGG were found to have the most significant treatment response associated with LITT, yielding a mean tumor reduction of 90% and a complete response rate of 36% at a mean follow-up of 24 months [25].

In addition to treating primary LGG, LITT has shown promise in the management of RN [26,27,28,29]. Studies investigating LITT for RN have shown significant local control, from 91% at 12 weeks, to 87.4% at 18 months [26,27]. To date, while non-invasive advanced imaging methodology such as perfusion magnetic resonance imaging (MRI) to differentiate tumor recurrence from RN is routinely used, histopathological examination remains the gold standard. As clinical outcomes may vary according to the underlying pathology, making a differentiation can provide some survival benefit [26,27]. LITT is unique in its ability to offer biopsy and thermoablative therapy during the same procedure, which can treat both RN and tumor. Further, LITT has been shown to decrease the need for corticosteroid use in some patients with RN, in which steroid dependence may be as debilitating as the tumor itself [26,27,30].

To enhance the current knowledge base of LITT for the management of LGG, we sought to present our institutional experience of LITT for presumed recurrent or newly diagnosed LGG and RN in terms of efficacy, safety, and survival outcomes.

## 2. Methods

The present study is part of a single-institution retrospective case series of clinical and survival outcomes after LITT for multiple pathologies, with this study focused on LGG and RN. Informed consent was not required due to the retrospective nature of the study and low risk of patient identification. The study was approved by the institutional review board of St. Joseph’s Hospital and Medical Center in Phoenix, AZ, USA. Data were collected from the electronic medical record, and MRI was reviewed from a picture archiving and communication system (Merge, IBM). The NeuroBlate (Monteris Medical, Minnetonka, MN, USA) and Visualase (Medtronic, Minneapolis, MN, USA) laser ablation systems were used for all patients included in this study. This case series has been reported in line with the PROCESS Guideline [31].

### 2.1. Inclusion and Exclusion Criteria

Patients treated with LITT for LGG or RN between 1 January 2013, and 31 December 2020, at Barrow Neurological Institute (St. Joseph’s Hospital and Medical Center, Phoenix, AZ, USA) by a single surgeon (K.A.Smith) were included in this study. Patients were excluded from the study if they were lost to follow-up immediately after the procedure or if the original diagnosis was not a new or recurrent LGG. Patients were either included in the LGG group or RN group based on the histological analysis of the intraoperative tissue biopsy. Progression after LITT was determined radiographically. Indications for the LITT surgery are discussed later in the manuscript.

### 2.2. Lesion Volume Estimation

Stereotactic preoperative T1-weighted gadolinium-enhanced and T2-weighted fluid-attenuated inversion recovery (FLAIR) MRIs were reviewed. The MRI data were used to measure lesion volumes. The lesion volume was determined by manual segmentation of cross-sectional areas, which were summed to compute the total lesion volume.

### 2.3. Statistical Analysis

Frequencies or means with standard deviations (SD) were used to describe cohort, lesion, and treatment characteristics. Median with interquartile ranges (IQR) were used to describe survival outcomes. Data for continuous variables are presented as mean (SD), and data for categorical variables are presented as frequency (percentage). Unpaired two-tailed *t* tests with significance set at *p* < 0.05 were used to compare means or medians between groups. Fisher’s Exact test and Pearson’s Chi Square test were used to compare categorical values, such as frequencies between groups. Kaplan–Meier analyses were used to generate the survival functions, and Log Rank (Mantel-Cox) analyses were performed to determine any significant difference between the survival curves with significance set at *p* < 0.05. SPSS Statistics version 25 (IBM Corp., Armonk, NY, USA) was used for all analyses.

## 3. Results

### 3.1. Demographics and Clinical Characteristics

A total of 9 patients with 14 radiographic lesions were identified as having undergone LITT therapy for management of radiographically presumed LGG (Table 1). The mean (SD) patient age at the time of LITT treatment was 50 (16) years, and 78% were men (*n* = 7) (Table 2). Three (33%) patients had a preoperative diagnosis of oligodendroglioma, WHO grade II, 2 (22%) diffuse astrocytoma, WHO grade II, 1 (11%) oligoastrocytoma, WHO grade II, 1 (11%) ganglioglioma, and 2 (22%) had newly progressive radiographic lesions without prior biopsy. Six (43%) lesions were in the frontal lobe, 3 (21%) peri-ventricular, 2 (14%) parieto-occipital, 2 (14%) thalamic, and 1 (7%) temporal (Table 2). Seven (50%) lesions were in the left hemisphere, 3 (21%) in the right hemisphere, and 4 (29%) were bilateral. All seven (83%) patients with a preoperative diagnosis of LGG had previous resection and among those, resections were performed a mean (SD) 1.6 (1.2) times. Treatment modalities prior to LITT therapy included iMRT in 6 (67%) patients, chemotherapy in 6 (67%), and SRS in 4 (44%). Two (22%) patients harboring inoperable lesions received LITT as the frontline modality for diagnosis and treatment. Seven (78%) patients received 3 or more treatment modalities prior to LITT. The mean (SD) time from histological diagnosis to LITT procedure was 13.2 (9.0) years for those with recurrent lesions, and 5.8 (0.2) years for those being treated frontline with LITT.

### 3.2. Indications for LITT

Most patients (*n* = 8, 89%) in this series were found to have new or progressive nodular intracranial enhancement on surveillance MRI that was concerning for recurrent tumor or reactive changes related to prior radiation treatment. Two (22%) patients had new symptoms, including a seizure and intermittent episodes of speech arrest. The two (22%) patients with new symptoms were then evaluated with MRI, which revealed new nodular intracranial enhancement concerning for recurrent tumor in one and no significant radiographic findings in the other. Major indications for LITT included tumors involving eloquent regions, multiple failed treatments, and poor functional status.

In 4 (44%) patients, advanced imaging with perfusion MRI was undertaken to determine preoperatively whether tumor recurrence or RN was more likely. Three (75%) of them had findings of decreased relative cerebral blood volume within the suspicious lesion, and while 2 (66%) perfusion MRI studies were consistent with the histopathological diagnosis of RN, one (33%) revealed itself as recurrent tumor on histopathology. The other one (25%) had findings of increased relative cerebral blood volume within the suspicious lesion but was identified as RN on histopathology.

### 3.3. Molecular Markers

Prognostic molecular markers were available for a subset of patients in this study due to the standard of laboratory practice during the study interval (Table 1). Of the 7 patients with available information on *IDH1* status, 2 (22%) were identified as having a wild-type status of *IDH1*.

### 3.4. Lesion Volume and Treatment Parameters

Volumetric measurements yielded a mean (SD) lesion volume of 4.1 (6.5) cm^3^ in preoperative T1-weighted gadolinium-enhanced MRI sequences, and a mean (SD) lesion volume of 26.7 (27.9) cm^3^ in T2-weighted FLAIR sequences (Table 3). Additional technical information, including the total energy delivered (kJ), laser on time (minutes), and total number of pulses became available for procedures performed after 14 December 2016. Thus, we report this data for a total of 4 patients who received treatment after this date. The mean (SD) total energy emitted from the LITT laser was 7.5 (9.5) kJ, with a mean (SD) number of 306 (400) pulses. The mean (SD) laser-on time, defined as the time that the LITT laser foot pedal was activated per patient, was 9.8 (13.5) min.

### 3.5. Clinical Outcomes

While there were no intraoperative complications, three (33%) patients were reported to have postoperative complications; two were permanent while the other one was transient. For the permanent complications, one patient had no complications peri-operatively, but was noted at a clinical follow-up one year later to have developed thalamic pain syndrome from the procedure; another patient developed mild diplopia with ptosis caused by oculomotor and trochlear cranial nerve palsies, both of which improved with rehabilitation but had not returned to baseline status during a one-year follow-up visit. For the transient complications, one patient formed a subdural hematoma requiring evacuation with a subdural drain placement and resolved by their hospital discharge (Table 1).

The mean (SD) length of hospital stay was 2.7 (2.0) nights. Histopathological evaluation of the biopsy obtained intraoperatively revealed RN in 4 (44%) patients, oligodendroglioma, WHO grade II in 2 (22%) patients, astrocytoma, WHO grade II in 2 (22%) patients, and anaplastic oligodendroglioma, WHO grade III in 1 (11%) patient (Table 1). The mean (SD) Karnofsky Performance Score was 80 (15) at preoperative screening, 76 (9) at first clinical follow-up, and 73 (15) at last clinical follow-up, which was not a significant decline (*p* = 0.09) (Table 4). At the end of the 8-year study period, 2 (22%) patients were deceased. The mean (SD) time from the date of operation to the last clinical follow-up was 35 (23) months.

### 3.6. Survival Outcomes

Survival outcomes were calculated for all patients in the cohort and separated based on the intraoperative histological diagnosis of either RN or histological subtype of recurrent or newly diagnosed tumor (Table 4). Of note, only one patient had an upgrade of histopathology from WHO grade II oligodendroglioma to WHO grade III anaplastic oligodendroglioma. All others either remained at the same grade of LGG or had RN (Table 1).

**Figure 1:**
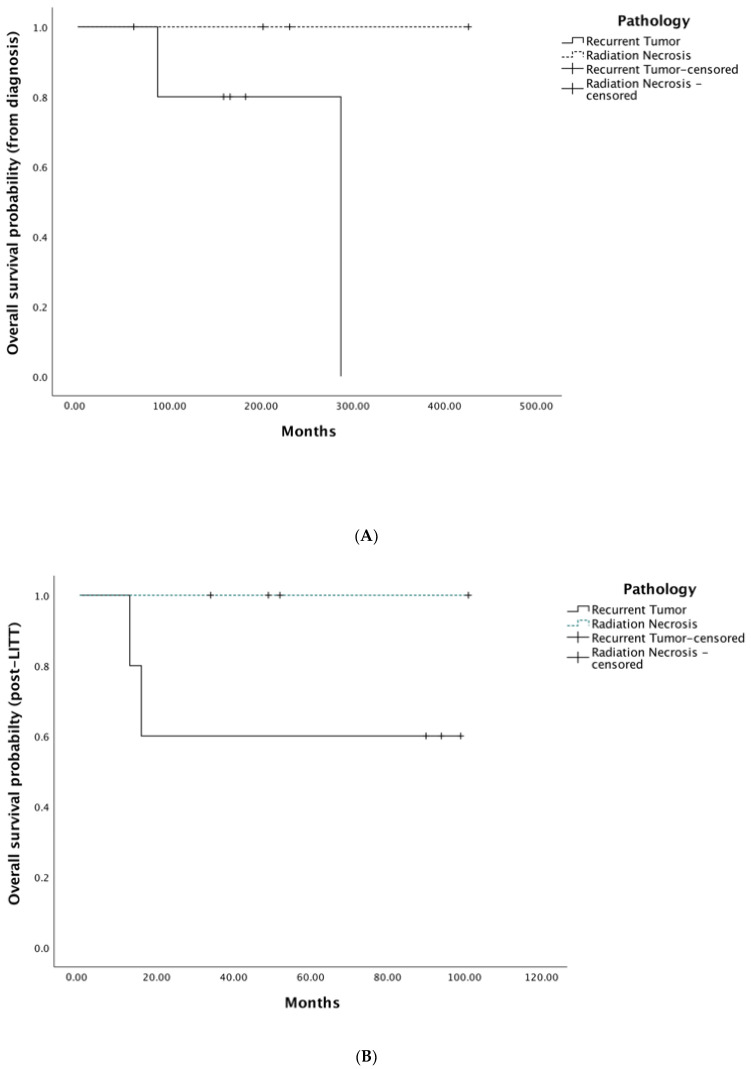

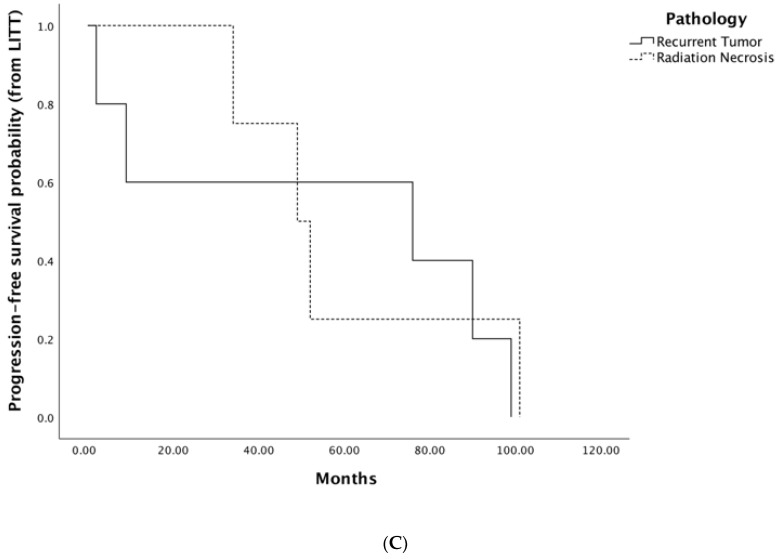
Kaplan–Meier survival analyses for 9 patients with low-grade glioma (LGG) or radiation necrosis (RN) treated with laser interstitial thermal therapy (LITT). (**A**) Cumulative overall survival from time of diagnosis. (**B**) Cumulative survival after treatment with LITT. (**C**) Progression-free survival after treatment with LITT.

The PFS from LITT was determined by radiographic evidence of progression of the LITT-treated lesion. Progressions were defined as new nodular enhancement at the laser ablation cavity on T1-weighted MRI enhanced by gadolinium. OS from LITT and from histological diagnosis were determined, respectively for patients that had passed away by the time of the survival analysis. Imaging was either taken routinely following a surveillance protocol or taken on an as needed basis if patients presented with progressive or new symptoms. All patients were included in the survival statistics. Kaplan-Meir analyses were also performed for the following two groups: RN and recurrent or newly diagnosed tumor (all histopathologies) (Figure 1A–C). Patients were marked as censored in the OS plots if they were alive at the time of the study. There were no significant differences between the survival distributions of RN versus biopsy proven tumor for the PFS (*p* = 0.72), OS from diagnosis (*p* = 0.2), and OS from LITT (*p* = 0.47). Of the two (22%) deceased patients, one deceased in hospice, while the other one presented to our emergency department with altered consciousness and recurrent falls, suggesting brain edema and subsequent herniation as the cause of death.

## 4. Discussion

In this single institutional case series, a total of 9 LGG patients were identified as having undergone LITT treatment for management of recurrent and newly diagnosed LGG during the 8-year study period. While current safety and efficacy evaluations of LITT for LGG are limited to small case series and case reports [11,24,32,33,34,35,36,37,38,39,40,41,42,43,44,45,46], we present one of only two studies reporting on survival outcomes in a larger-scale contemporary adult case series [24].

### 4.1. Safety and Efficacy of LITT

Our data suggest that LITT may be safely applied for the treatment of LGG, albeit the procedure carries a risk of potential complications of varying severity. In this case series, 33% of patients experienced post-procedural complications, two of which were permanent (cranial nerve palsies, thalamic pain syndrome), and one of which was transient (acute subdural hematoma). Therefore, the true long-term complication rate is 22% in our study, representing an acceptable amount of risk associated with the LITT procedure. Interestingly, both permanent complications were associated with lesions in thalamic structures, whereas all other lesions had either no complications or only transient ones. Some of these observed complications are in accordance with what has been previously reported by Jethwa and Pruitt et al. during their initial experience with LITT. The two groups identified common complications of LITT as hemorrhage from avulsion of an artery, brain edema in relation with large lesions, thermal injury to eloquent structures, and malposition of the catheter [11,47]. Transient neurological deficits and pneumocephalus have also been described [47,48,49]. In a large-sample cohort study comprising 102 LITT applications, Patel et al. observed postoperative neurological deficits in 13.7%, of which 64.3% resolved at one-month follow up, but also involved two perioperative deaths secondary to refractory edema after the LITT procedure [38].

This study demonstrated an association between LITT treatment of eloquent lesions and permanent neurological complications in two patients. To address unfavorable outcomes in patients with eloquent tumors, Del Bene and colleagues proposed a novel concept of integrating preoperative magnetoencephalography and diffusion tensor imaging, and intraoperative neurophysiologic monitoring with the aim of reducing the procedural morbidity [50]. The advantages of this synergistic approach are manifold: While continuous intraoperative neuromonitoring facilitates real-time tracking of neuronal damage, preoperative motor planning enables identification of the most appropriate trajectory and informs on proximity to eloquent structures and on the extent of ablation that may be safely applied. Similarly, Luedke et al. reported on neuromonitoring-guided LITT for mesial temporal lobe ablation in two patients with medically refractory epilepsy [51]. Collectively, these reports demonstrate that use of auxiliary pre- and intraoperative technologies may increase the safety profile of LITT for high-risk lesions.

The Karnofsky Performance Score is a widely used tool in oncology and a number of other disciplines to assess the quality of life and physical condition of patients on a scale from 100% (full function) to 0% (death) based on the performance of activities of daily living [52,53]. The baseline functional status of our LGG cohort was relatively high, with a mean KPS of 80% at preoperative evaluation, and although the KPS decreased to 76% at the first clinical follow-up and to 73% at the last clinical follow-up, this trend was not statistically significant (*p* = 0.09). We conclude that the patients’ functional status was grossly preserved throughout the post-procedural phase and long-term follow-up, a criterion that is of paramount value for patients and their families when making treatment-based decisions.

### 4.2. LITT Treatment Planning

There are some unique aspects worth considering when designing a LITT treatment plan, such as the timing and the previous treatment modalities received. In this cohort, LITT treatment was carried out 11.6 years after receiving the initial diagnosis. While the time from diagnosis to LITT remains a poorly elucidated parameter, we aim to provide a launching point for future considerations for LITT treatment planning. Further, all patients with recurrent lesions (*n* = 7, 78%) had undergone previous surgical resection, and some patients had additional IMRT (69%), SRS (44%), and chemotherapy (67%). All patients with recurrent tumors underwent three or more treatments prior to LITT. These findings provide evidence that LITT applications most consistently serve as salvage therapies for patients unable to tolerate surgery, having failed multiple treatments, or harboring inoperable lesions. However, 2 patients (22%) in this cohort did not receive any treatment prior to LITT, including surgical resection. Justifying the use of LITT as the frontline therapy in select patients involves individualized decision-making, accounting for tumor biology, growth behavior, and patient preference. In a retrospective analysis comprising 34 high-grade gliomas, LITT was delivered as upfront therapy in 19 and as salvage therapy in 16 patients, without further comparisons between the two strategies being provided [54]. While our investigations aim at advancing the current treatment strategies of LITT, recommendations of its superiority as a salvage, supportive, or frontline therapy cannot be concluded at this time.

### 4.3. LITT Survival Outcomes

Current literature on LITT treatment for LGG, especially on a larger-scale basis, remains scarce. However, the few studies that are available report similar survival rates as reported by our study. Using LITT in a multimodal context, Leonardi et al. determined a mean OS of 144 months after diagnosis and a mean OS of 34 months after LITT in 7 recurrent low-grade astrocytoma patients [24]. While the study reports a mean PFS of 16 months after LITT, the median PFS in this study was significantly higher at 49 months in our pooled analysis and 50 months in our low-grade astrocytoma patients. Further, the pooled median OS from diagnosis in this study was slightly higher, at 173 months, suggesting that LITT may have been adopted later within the disease course. In addition, Leonardi et al. observed a high-quality functional status at 11, 20, 21, 33, and 43 months in 5 low-grade astrocytoma patients, which in conjunction with our non-significant decline in KPS suggests LITT as a beneficial adjunct to preserve the quality of life in LGG patients while providing local disease control.

In the pediatric population, Pehlivan et al. assessed the application of LITT as safe and efficient in 4 children with LGG whose seizure presentations either improved significantly or resolved entirely. Among other tumor entities included in the study, LITT yielded the most significant effects in LGG [25]. A case series of 8 children with LGG reported significant cytoreductive effects 15 to 36 months after LITT ablation [55].

### 4.4. LITT Survival Outcomes in Context

Although LITT may be used as the frontline therapy for inoperable lesions, it most commonly is applied as salvage therapy for recurrent disease. Therefore, we sought to compare LITT with competing focal treatments such as SRS or iMRT for recurrent or progressive LGG. However, such discussion is limited by either the inclusion of LGG in pooled studies mainly comprising of high-grade tumors [56], or assessment of SRS and iMRT in multimodal context for newly diagnosed LGG [57] and in children [23,58], which largely limits the comparability with our cohort.

For primary LGG treatment, surgical resection is considered the most efficient treatment modality when rapid mass reduction and symptom control are desired [59]. Generally, maximized and early surgical resection are associated with favorable survival outcomes [17,60,61,62,63,64,65,66]. The reported 5- and 8-year OS were 97% and 91%, respectively, with an at least 90% extent of resection [17]. Patel et al. determined a median OS ranging from 6.3 years in *IDH*-wildtype LGG to 16.5 years in *IDH*-mutant LGG [62]. Jakola et al. reported a median OS of 5.8 years and 14.4 years upon late and early surgical resection, respectively [60]. While these findings advocate for early and maximal primary surgical resection of LGG, this treatment modality takes a backseat when involving eloquent structures, opening opportunities for salvage treatments such as SRS, iMRT, chemotherapy, and LITT [59].

Although radiotherapy was long considered the mainstay adjunct after surgical resection [67,68], its initiation at primary versus recurrent glioma presentation remains controversial. A randomized clinical trial lead by van den Bent et al. reported a significantly lengthened median PFS of 5.3 years in patients who received postoperative early radiotherapy compared to 3.4 years in the control group (*p* < 0.0001), without a significant difference in overall survival (7.2 vs. 7.4 years, *p* = 0.872) [69]. Although these PFS and OS rates are favorable over the ones presented by LITT, in addition to being a non-invasive procedure, radiotherapy has been associated with permanent side effects, the most common of which are RN, cognitive impairment, and secondary malignancies [9,10].

Multiple studies have assessed the efficacy of combination radio-chemotherapy in LGG [70,71,72,73]. Among those, temozolomide was associated with a PFS of 76% at 6 months and 39% at 12 months, while achieving a high response rate in 47% [72]. Combined radio-chemotherapy with PCV (procarbazine, CCNU, vincristine) demonstrated longer median OS of 13.3 years compared to radiotherapy alone, with a median OS of 7.8 years (*p* = 0.003) [71]. In summary, these findings suggest that LGG should be treated in a multimodal context, considering the significantly prolonged OS rates achieved through primary surgical resection, radiation, and combined radio-chemotherapy.

### 4.5. Limitations

This study has multiple limitations owing to its retrospective study type and the limited number of qualifying participants. In the recent years, LITT has emerged as a relatively novel strategy whose efficacy and safety thresholds are yet to be determined, particularly in LGG. Practicing neurosurgeons are committed to carefully select LITT-applicable patients in an era where a detailed understanding of this modality’s efficacy remains obscure, and this provides an explanation for the limited availability of select patients. Furthermore, the novelty of LITT eventuates in a lack of clear indications for LITT and a clinical baseline heterogeneity among patients, contributing to the limitations of this study. Secondly, this cohort is limited by significant heterogeneity, such that patients had received various prior treatments, with LITT being initiated at varying time points thereafter, and with intentions of both salvage and frontline therapy. Additionally, molecular information was inconsistently available within the early interval of the study. Despite these limitations, the present study serves as a launching point for future investigations of LITT’s efficacy, safety, and survival outcomes in patients with recurrent or newly diagnosed LGG.

## 5. Conclusions

LITT is a safe and effective treatment option for management of recurrent LGG and RN, however, within this series it is associated with a moderately high immediate postoperative complication rate. Further, this study outlines LITT as a minimally invasive salvage therapy for the management of LGG, benefiting patients who have had multiple failed treatments or are unable to tolerate an additional resection. Its role as a frontline therapy requires a dedicated study for select patients who are unamenable to primary surgical resection.

## Figures and Tables

**Table 1:** Individual demographic, lesion, and treatment characteristics for 9 patients treated with laser interstitial thermal therapy (LITT) for radiographically presumed recurrent or newly diagnosed low-grade glioma (LGG) and radiation necrosis (RN).

No.	Age	Sex	Original Diagnosis	Number of Lesions	Side and Location	Number of Previous Resections	Previous SRS	Previous iMRT	Previous Chemotherapy	Intraoperative Histology	*IDH1* Status	Complications	Evidence of Radiographic Progression after LITT	PFS (Months)	Clinical Status at Time of Study
1	41	M	Ganglioglioma	2	Bilateral frontal/peri-ventricular	3	Yes	No	No	RN	n/a	None	No	101	alive
2	51	M	Oligodendroglioma, grade II	2	Bilateral frontal/periventricular	1	No	Yes	Yes	Recurrent oligodendroglioma, grade II	n/a	None	No	99	alive
3	50	M	n/a	1	Left thalamic	0	No	No	No	Oligodendroglioma, grade II	Mutation	Post-operative, permanent; thalamic pain syndrome	Yes	76	alive
4	32	F	n/a	1	Left thalamic	0	No	No	No	Astrocytoma, grade II	Mutation	Post-operative, permanent; CN III/IV palsies	No	90	alive
5	61	M	Oligoastrocytoma, grade II	2	Left frontal/peri-ventricular	3	Yes	Yes	Yes	RN	Mutation	None	No	52	alive
6	65	M	Astrocytoma, grade II	1	Left frontal	2	Yes	Yes	Yes	RN	Mutation	None	No	49	alive
7	24	M	Astrocytoma, grade II	2	Left frontal/parieto-occipital	3	No	Yes	Yes	Recurrent astrocytoma, grade II	Wildtype	None	Yes	9	deceased
8	55	M	Oligodendroglioma, grade II	2	Right frontal/temporal	1	Yes	Yes	Yes	Anaplastic oligodendroglioma, grade III	Wildtype	None	Yes	2	deceased
9	72	F	Oligodendroglioma, grade II	1	Right parieto-occipital	1	No	Yes	Yes	RN	Mutation	Post-operative, transient; acute subdural hematoma	No	34	alive

**Table 2:** Demographics and lesion characteristics of 9 patients with low-grade glioma (LGG) and radiation necrosis (RN) treated with laser interstitial thermal therapy (LITT).

Characteristic	Patients (*n* = 9) or Lesions (*n* = 14)
Age, mean (SD), years	50 (16)
Sex	
Male	7 (78)
Female	2 (22)
Cerebral location, lesions	
Frontal	6 (43)
Periventricular	3 (21)
Parieto-occipital	2 (14)
Sub-cortical (thalamic)	2 (14)
Temporal	1 (7)
Cerebral hemisphere, lesions	
Left	7 (50)
Right	3 (21)
Bilateral	4 (29)
Number of previous resections (SD)	1.6 (1.2)
Time from original diagnosis to LITT, mean (SD), years	11.6 (8.5)
Treatment of recurrent lesions	13.2 (9.0)
Treatment of primary lesions	5.8 (0.2)
History of radiation therapy	
IMRT	6 (67)
SRS	4 (44)
History of chemotherapy	6 (67)
History of resection	7 (78)
≥3 previous treatments	7 (78)

Data are no. (%) of patients unless otherwise indicated. IMRT, intensity-modulated radiation therapy; SRS, stereotactic radiosurgery.

**Table 3:** Lesion volumes and treatment parameters of 9 patients with low-grade glioma (LGG) and radiation necrosis (RN) treated with laser interstitial thermal therapy (LITT).

Variable	Patients (*n* = 4)
Enhancing T1-weighted lesion volume, cm^3^, mean (SD)	4.1 (6.5)
Fluid-attenuated inversion recovery lesion volume, cm^3^, mean (SD)	26.7 (27.9)
Total energy delivered, kJ, mean (SD)	7.5 (9.5)
Number of pulses, mean (SD)	306 (400)
Laser on time, min, mean (SD)	9.8 (13.5)

Data are no. (%) of patients unless otherwise indicated.

**Table 4:** Survival analysis and clinical outcomes of 9 patients with low-grade glioma (LGG) and radiation necrosis (RN) treated with laser interstitial thermal therapy (LITT).

Variable	Oligodendroglioma, WHO Grade II (*n* = 2)	Astrocytoma, WHO Grade II (*n* = 2)	Anaplastic Oligodendroglioma, WHO Grade III (*n* = 1)	Radiation Necrosis (*n* = 4)	Pooled (*n* = 9)
Progression-free survival from LITT, mo, median (IQR)	88 (12)	50 (41)	2 (n/a)	51 (19)	52 (56)
Overall survival from LITT, mo, median (IQR)	97 (3)	110 (16)	16 (n/a)	501 (21)	52 (60)
Overall survival from diagnosis, mo, median (IQR)	175 (9)	123 (36)	287 (n/a)	217 (120)	183 (72)
Karnofsky Performance Score, mean (SD)					
Preoperative	90 (0)	85 (7)	70 (n/a)	75 (10)	80 (15)
First clinical follow-up	80 (14)	75 (7)	70 (n/a)	75 (10)	76 (9)
Last clinical follow-up	80 (14)	65 (21)	50 (n/a)	80 (8)	73 (15)
Time to last clinical follow-up, mo, mean (SD)	38 (36)	38 (25)	15 (n/a)	37 (26)	35 (23)
Clinical status at end of study period					
Deceased	0 (0)	1 (50)	1 (100)	0 (0)	2 (22)
Alive	2 (100)	1 (50)	0 (0)	4 (100)	7 (78)

Data are no. (%) of patients unless otherwise indicated.

## Data Availability

Data available within the article.

## Abbreviations

FLAIR: fluid-attenuated inversion recovery; IDH, isocitrate dehydrogenase; IQR, interquartile ranges; KPS, Karnofsky Performance Score; LGG, low-grade glioma; LITT, laser interstitial thermal therapy; iMRT, intensity-modulated radiation therapy; MRI, magnetic resonance imaging; OS, overall survival; PFS, progression-free survival; RN, radiation necrosis; SD, standard deviation; SRS, stereotactic radiosurgery; WHO, World Health Organization.
